# Polymeric nanocarrier systems for photodynamic therapy

**DOI:** 10.1186/2055-7124-18-19

**Published:** 2014-12-08

**Authors:** Li Li, Kang Moo Huh

**Affiliations:** Department of Polymer Science and Engineering, Chungnam National University, 99 Daehak-ro, Yuseong-gu, Daejeon, 305-764 Republic of Korea

**Keywords:** Photodynamic therapy, Photosensitizer, Drug delivery, Polymer, Nanocarrier, Conjugates

## Abstract

Photodynamic therapy (PDT) is an emerging treatment modality that involves the combined action of photosensitizers (PSs) and light for treatment of solid tumor and other diseases. Although this therapeutic method has been considered as an alternative to classical cancer treatments, clinical PDT requires further advances in selectivity and therapeutic efficacy to overcome numerous shortages related to conventional PDT. In this regard, great efforts have been devoted to the development of polymeric nanocarrier-encapsulated PSs for targeted PDT, aiming at improvement of water solubility and tumor-specificity of hydrophobic PSs. Here, we discuss the general concepts and considerations of polymeric nanocarriers for efficient delivery of PSs. In recent, the amphiphilic PS-polymer conjugate-based self-quenchable nanoparticles and PS-polymer-conjugate/quencher nanocomplexes have emerged as an attractive delivery platform for efficient and reliable PDT. They can incorporate and deliver the PS in a photodynamically inactive state but demonstrate cytotoxic effects by tumor environment-sensitive activation mechanisms, so that the photodynamic cancer treatment can achieve maximum target specificity. Here, we report the recent achievements on the development of activatable PS formulations based on PS-polymer conjugates.

## Introduction

Photodynamic therapy (PDT), a light-activated chemotherapeutic treatment, has emerged as an innovative clinical modality for tumors and nononcological diseases [1, 2]. This modality is based on the selective retention of a previously administrated photosensitizing molecule in a target site and a measured light dose of appropriate wavelength is then used to irradiate the target tissue. Upon light irradiation, the photosensitizing molecule interacts with molecular oxygen to generate various reactive oxygen species to damage target cells via apoptosis and necrosis [2, 3]. PDT offers several advantages over conventional therapies for malignant diseases [4–6]. For example, PDT is a minimally invasive method that destroy target cells without surgical risks, serious damages, and systemic complications. Since PSs are typically harmless without light, tumor site treatment can be precisely targeted by selective illumination, thus, PDT is highly selective and targeted in action. PDT can be applied repeatedly without initiating resistance or exceeding total dose limitation (as associated with radiotherapy). Over the past decade, PDT is gradually becoming a more widely used clinical technique and has received regulatory approval for treatment of a number of solid tumors [7], such as lung [8], bladder [9], head and neck [10], ovarian [11], prostate [12], skin [13] and bone carcinomas [14].

**Figure 1:**
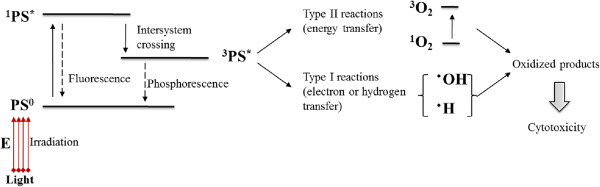
**Mechanism of PDT cytotoxicity: photophysical and photochemical reactions represented by modified Jablonski diagram.**

The photosensitizing molecules, named as photosensitizers (PSs), function as catalysts when they absorb visible light and convert molecular oxygen to a range of highly reactive oxygen species (ROS) (singlet oxygen and free radicals, such as OH^-^, O_2_^2-^ and O_2_^-^). The detailed mechanism of action of PDT using PS is illustrated as shown in Figure 1 [7, 15]. Briefly, PS has a stable electronic configuration with a singlet state in their lowest or ground state energy level (PS^0^). Upon activation, the PS in its ground state absorbs a photon and is promoted into an excited singlet state (^1^PS^*^). The excited singlet state can relax back to ground state by emitting a fluorescent photon or can convert to the triplet state (^3^PS^*^) via intersystem crossing which involves a change in the spin of an electron. This triplet state is a photoactive state, which can interact with molecular oxygen and produce reactive oxygen species by undergoing two main reactions, Type I and Type II reactions. In Type I reaction, the PS transfers an electron to various receptor molecules, producing free radicals or superoxide ions resulting from hydrogen or electron transfer. Type II reaction leads to produce the electronically excited and highly reactive state of oxygen known as singlet oxygen. In PDT, Type II processes are most relevant, and the generation of singlet oxygen is responsible for the irreversible damage of tumor cells [16]. In addition to directly killing tumor cells through production of ROS under light irradiation [17], PDT also can damage the tumor-associated vasculature leading to tumor infarction [18], and can activate the immune response against tumor cells [19, 20].

In a PDT process, PSs are critical to the successful eradiation of malignant cells. An ideal PS should meet several crucial requirements [21]: (i) identified purity and composition; (ii) minimal dark toxicity; (iii) photostability; (iv) strong absorbance in a near-IR spectrum range with high extinction molar coefficient; (v) water solubility; (vi) tumor site target specificity; (vii) adequate clearance rate from the body. The first generation PS refers to Hematoporphyrin (Hp) and Photofrin^®^ (hematoporphyrin derivative, HpD) [22]; and, Photofrin is the first PDT agent approved for clinical use. It has been considered as a therapy against various cancers, such as lung, esophageal bladder, brain, breast, and early-stage cervical cancer. However, although Photofrin has demonstrated significant therapeutic effects, it still suffers from several drawbacks: first, Photofrin is a complex and undefined mixture of dimeric and oligomeric compounds having poor tissue penetration due to its relatively weak absorbance in the red region of the spectrum; second, it has a poor selectivity in terms of target tissue/healthy tissue ratios; third, it has a low molar extinction coefficient that requires use high doses of Photofrin and light for adequate tumor eradication; forth, it readily accumulate and stay in skin for a longtime, causing long-lasting cutaneous photosensitivity [23]. To address these problems, a wide variety of second generation PSs, such as porphyrin derivatives [24, 25], phthalocyanines [26–28], and chlorins [29, 30], have been developed. Comparing to the first generation, they have the advantage of being pure and well characterized; they can effectively generate singlet oxygen and have absorption maxima at wavelength longer than 630 nm, at which light penetration in tissue is enhanced; their relative high selectivity for malignant sites and fast elimination from the body leads to a reduced skin photosensitivity [22].

Although second generation PSs have addressed several problems with first generation PSs, they still face the challenges associated with the PS delivery [31]. Most existing second generation PSs are aromatic and hydrophobic in nature with poor or limited solubility in water and hence difficult to be intravenously administrated. Even in the case of water-soluble PSs, the poor accumulation selectivity at malignant sites makes them far from clinical applications. Formulating hydrophobic PSs using lipidic (e.g. Cremophor) or organic (e.g. propylene glycol) excipients is reasonable for topical or local administration, but can cause unpredictable biodistribution, toxicity, and hypersensitivity if administrated intravenously [15]. The unpredictable biodistribution can lead to a high plasma retention and unexpected accumulation of PSs in healthy tissues like skin, which often results in accidental damage on blood vessels or non-disease tissues when the optical equipment applied, and/or skin photosensitivity when the patients expose to the sunlight or strong indoor lights. In addition, as for excipients like Cremophor, the issues with allergy, hypersensitivity reactions, and nephrotoxicity have been often reported [32, 33]. Therefore, a challenge in PDT for treatment of malignant diseases is to design a safe and tumor-specific carrier platform for systemic delivery of PSs.

**Figure 2:**
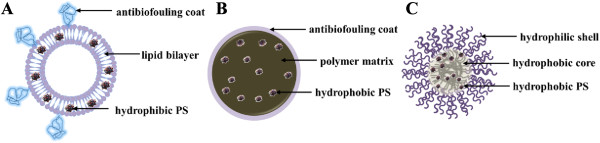
**A schematic diagram of PS-encapsulated polymeric nanocarriers: (A) liposome, (B) polymeric nanoparticle, and (C) polymeric micelle.**

**Figure 3:**
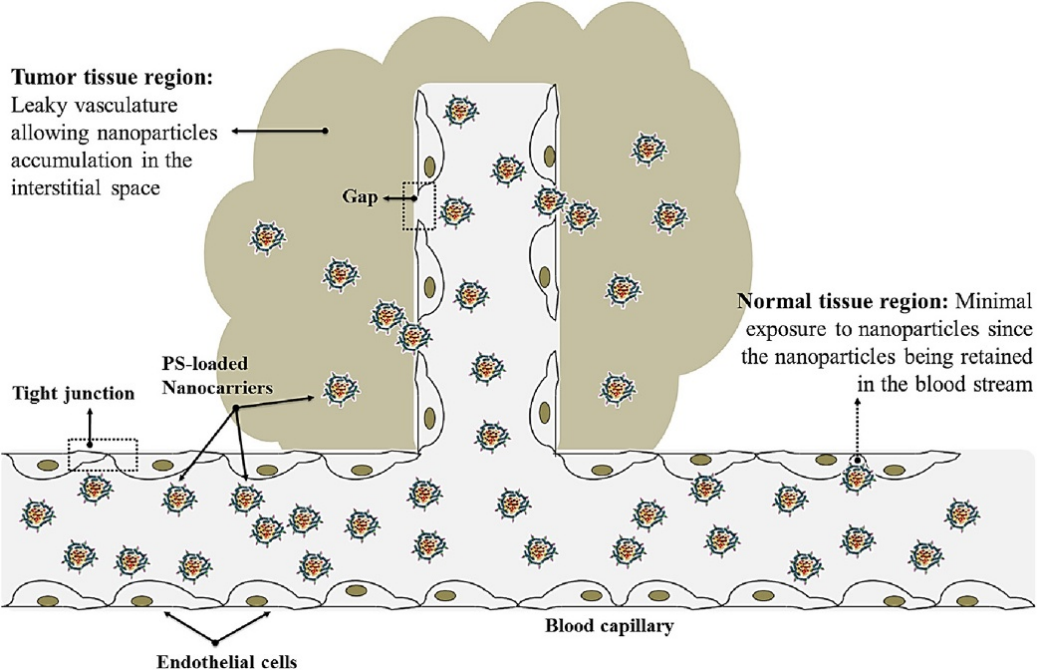
**Schematic presentation of passive targeted PDT through EPR effect.** PSs encapsulated into nanocarriers can reach tumors selectively through the “leaky” vasculature surrounding the tumors.

To date, a variety of macromolecular nanocarrier platforms such as liposomes [34–36], polymeric nanoparticles [37–41], and micelles [42–45] have been investigated for their potential application in PS delivery (Figure 2), which can provide an effective solution to overcome the shortages of current PSs associated with intravenous administration and selective delivery of the PS to the tumor sites. Beneficial effects of these nanocarriers lie in their excellent colloid dispersity in water that enables solubilization of hydrophobic PS in physiological condition and the enhanced accumulation at tumor sites through enhanced permeability and retention (EPR) effect (Figure 3) [46, 47]. Generally, PS can be encapsulated using these carriers by both physical methods using hydrophobic or electrostatic interactions between PS and polymers and chemical methods using various conjugation reactions of PSs to polymers and nanoparticles. The general concepts and considerations of polymeric nanocarriers for delivery of PS will be resumed. In addition to the delivery strategies to modulate the pharmaceutical features and biodistribution of PS, a series of amphiphilic PS-polymer conjugate-based self-quenchable nanoparticles [48–52] and PS-polymer-conjugate/quencher nanocomplexes [53–55] have been recently developed. A common characteristic feature of these platforms is that they can incorporate and deliver the PS in a photodynamically inactive state and create active forms and produce cytotoxic effects only at the tumor site. Since the direct tumor cell destruction essentially depends on the in situ generation of cytotoxic singlet oxygen, a controllable singlet oxygen production with high selectivity and localization would lead to more efficient and reliable PDT, thereby, significantly reducing the associated side effects in PDT, such as skin photosensitivity. In a second part, we will describe the strategies that involved in construction of activatable PS formulations based on polymer-PS conjugates and their applications in tumor site-specific PDT.

## Review

### Polymeric nanocarriers for delivery of hydrophobic photosensitizers

To enhance the water solubility and increase the specific accumulation of PSs at the target site, a generally used strategy is encapsulation of the PSs to macromolecular nano-constructs. In this respect, liposomes [34–36], polymeric micelles [42–45], and polymeric nanoparticles [37–41] have been extensively studied for serving as PS carriers in PDT. A common characteristic feature of above mentioned platforms is that they demonstrate to show tumor-selective accumulation due to the enhanced microvascular permeability and impaired lymphatic drainage in the tumor tissue, a phenomenon which Maeda et al. termed the EPR effect [56, 57]. Therefore, systemic PS delivery based on polymeric nanocarrier systems can provide an effective way to not only enhance the water solubility but also modulate the biodistribution profile of hydrophobic PSs and thus facilitating intravenous administration and selective delivery of PS to target tissues.

### Liposomes

Extensive research has been carried out to use liposomes, bilayered phospholipid vesicles, to formulate the hydrophilic or hydrophobic PSs owing to their simple archetypal structures, controllable sizes, and convenient preparation procedure. The hydrophobic PS, such as porphyrin [58] and phthalocyanine derivatives [59], can be dissolved in the phospholipid bilayer region, while the water soluble molecules, such as the prodrug aminolevulinic acid [60], can be encapsulated in the inner core of liposomes. The advantageous of liposome carriers for PS delivery have been described by a number of investigations, which have shown their beneficial effects over other formulations such as simple aqueous dispersions of the PSs. For example, Richter et al. have studied biodistribution and clearance of benzoporphyrin derivative monoacid ring A (BPD-MA) encapsulated into a unilamellar dipalmitoylphosphatidyl-choline (DPPC) liposome in comparison to the drug dissolved in a dimethyl sulfoxide solution and a PBS solution [61]. The in vivo study in M1 rhabdomyosarcoma-bearing mice has shown that the maximum concentration of liposome formulation in tumor tissue was obtained 15 min post-injection against 3 h for both aqueous formulations. Under light treatment, the liposomal formulation showed significantly more therapeutic efficacy than both aqueous formulations. However, conventional liposomes (unmodified multilamellar or unilamellar liposomes composed of phospholipid) are sometimes suffered from short plasma half-life, which is insufficient time for tumor cell uptake given the rapid elimination by the reticuloendothelial system (RES) and decomposition due to in-body lipid exchange interactions [34]. In this regard, many approaches based on surface modifications of liposomes were explored to produce the long-circulating liposomes featuring substantially enhanced plasma stability. For instance, inclusion of lipids with poly(ethylene glycol) (PEG)-head groups can stabilize the liposome and increase its bioavailability by facilitating evasion of the RES. PEGylated liposomes loaded with chlorin e6 ester have been reported to enhance PDT efficiency compared to the free PS in gastric cancer cell lines in vitro and in vivo [62]. For the long-circulating liposomes, conjugation of the cancer cell targeting moieties, such as ligands, antibodies, proteins and peptides, can effectively increase cancer cell targeting specificity and improve the cellular uptake of liposomes [34, 63].

### Polymeric nanoparticles

As an alternative to liposomes, polymeric nanoparticles have been considered as prospective delivery vehicles for PS payloads in PDT. The particle size of polymeric nanoparticles can be easily tailored in the nanometer range by altering the polymer composition and manufacturing processes [64], which is an important factor that enable the PS formulations to be delivered to tumor site through EPR effect and prevent recognition by macrophages and proteins with prolonged circulation time in the blood. In addition, by coating with “stealth” PEG, the nanoparticles can efficiently avoid to be taken up by RES after intravenous administration and consequently increasing the blood circulation time [65, 66]. Usually, the hydrophobic PSs can be physically entrapped into nanoparticles by hydrophobic or electrostatic interactions between PS and the polymer. Biodegradable polymers, such as polyglycolide (PGA), polylatide (PLA), and their copolymer poly(_D,L_-lactide-co-glycotide) (PLGA), have been particularly utilized as matrix materials for PS delivery [40, 67]. The main advantages lie in their versatility, physical robustness, and high drug loading. In addition, their surface properties, morphologies, and composition of polymer matrices can be easily optimized to control the polymer degradation and drug release kinetics for achieving controlled release of PS. As reported by Allemann et al. [68], hexadecafluoro zinc phthalocyanine, was formulated in PEG-coated PLA nanoparticles by the salting out technique with a drug loading of 0.61%. As tested in EMT-6 tumour-bearing mice at a dose of 5 μmol/kg, the PS-loaded nanoparticles caused tumor regression in 100% of mice compared to only 60% with the Cremophor EL emulsion. The results indicated that formulation in the biodegradable nanoparticle improved PDT response of the tumor as compared to conventional Cremophor EL emulsion while providing prolonged tumor sensitivity towards PDT. A second generation PS, meso-tetra(hydroxyphenyl) porphyrin (p-THPP) was entrapped into sterile sub-150 nm biodegradable nanoparticles based on three selected polyesters PLGA (50:50 PLGA and 75:25 PLGA) and poly(D,L-lactide) (PLA) using the emulsification-diffusion technique [69, 70]. The effect of copolymer composition on particle size, drug loading efficiency, and PDT effect has been investigated. A follow-up study was carried out to assess the PDT efficacy of these p-THPP loaded nanoparticles on EMT-6 mouse mammary tumor cells as compared to free p-THPP. The relatively low drug concentration and the short incubation times of nanoparticles with cells required to induce satisfactory photodynamic damages demonstrated that p-THPP loaded nanoparticles offer superior photoactivity as compared to the free drug.

### Polymeric micelles

Polymeric micelles are macromolecular complexes formed spontaneously when amphiphilic block or graft copolymers are dispersed in aqueous solution above the critical micelle concentration (CMC). The hydrophobic segments of amphiphilic copolymer exhibit poor compatibility with the aqueous phase and readily assemble to form a core structure for incorporation of hydrophobic drugs. The outer shell region made up by the hydrophilic segments maintains a stabilizing interface between the hydrophobic compartment and the aqueous environment, thus, allowing to solubilize the hydrophobic PSs. In addition, formulating hydrophobic PSs with micelles also could facilitate the control of the drug release in specific site by diffusion, polymer degradation or micelle dissociation mechanisms. Large variety of lipophilic polymers can be used as core forming blocks, such as poly(amino acids) and poly(esters) [71]. However, the corona has almost exclusively been constituted from PEG because of its high water solubility, biocompatibility, and nonfouling properties [72]. Usually, physical incorporation of the hydrophobic PSs can be achieved by various manufacturing processes, such as thin-film hydration, dialysis, or oil-in-water emulsion methods, which lies on the hydrophobic-hydrophobic interaction between drugs and hydrophobic moieties of the copolymer. In PDT, polymeric micelle has been extensively investigated as an alternative platform for delivery of hydrophobic PS and presents several advantages, such as simple preparation, efficient drug loading without chemical modification, and controlled release. Such micelles also exhibit long blood-circulation times and tumor selectivity based on the EPR effect, thus decreasing the unfavorable biodistribution of hydrophobic PSs and consequently reducing the adverse effects like skin photosensitivity. Li et al. reported a formulation of PEG-b-poly (caprolactone) (PEG-PCL) micelle incorporated with hydrophobic protoporphyrin IX (PpIX) [73]. The micelles have a high PpIX-loading efficiency of 82.4% and a narrow size distribution with a mean diameter of 52 nm. In comparison with the free drug, formulation of PpIX in micelles enhanced the total intracellular accumulation of the agent, and thereby markedly increased the photocytotoxicity of PpIX. Hence, the formulation of PpIX in block copolymer micelles may allow the desired PDT efficiency to be achieved at a reduced dose of drug and/or light. The poly(2-ethyl-2-oxazoline)-b-PLA diblock copolymer micelle was used to incorporate meta-tetra(hydroxyphenyl) chlorin (mTHPC) [74]. The m-THPC-loaded micelles exhibited marked PDT effect in vivo, and the therapeutic efficacy was similar to free drug. However, the m-THPC-loaded micelles had less skin phototoxicity after an extended delivery time comparing with free drug. The enhanced tumor specificity of a PS-loaded polymeric micelle could improve therapeutic efficacy while decreasing its adverse effects. Unfortunately, most hydrophobic PSs incorporated into micelles easily form aggregates due to their π-π interactions and hydrophobic characteristics. Such aggregate formation may severely decrease singlet oxygen generation due to the self-quenching of the excited state. With this regard, incorporation of unaggregated monomeric molecules of PSs into polymeric micelles was considered to retain the therapeutic efficacy of PS’s micellar formulation. Knop et al. [75] has reported that the pheophorbide a (PhA)-loaded PEG-PCL micelle with 20 nm in hydrodynamic diameter, corresponding to approximately 200 molecules of polymer and 4 molecules of monomeric PhA per nano-object, was able to efficiently generate singlet oxygen in the medium. In vitro tests on human cancerous cells have revealed a ca. 2-fold enhanced photocytotoxicity and cellular uptake compared to free PhA.

**Figure 4:**
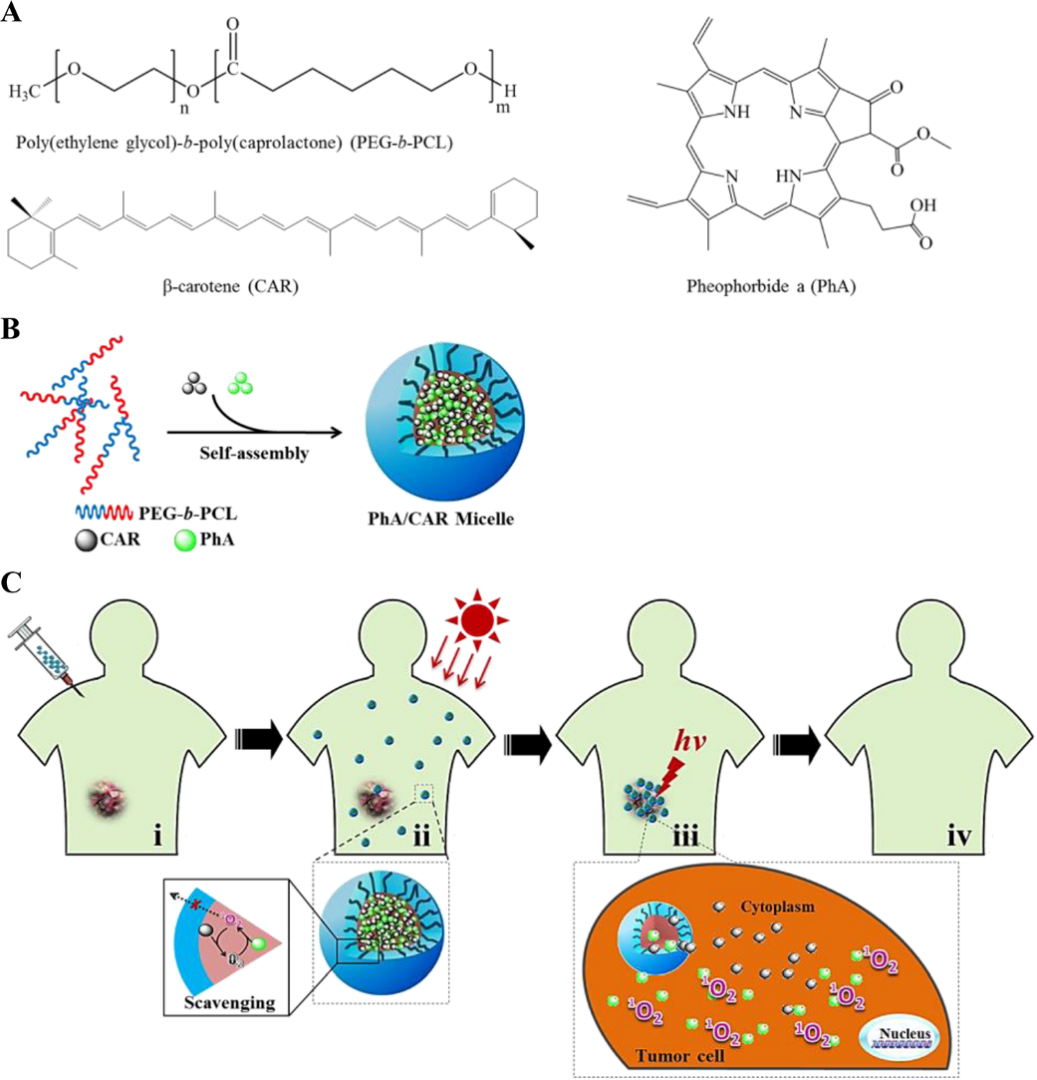
**PEG-PCL micelles co-loaded with PhA as the singlet oxygen generator and CAR as the singlet oxygen scavenger for targeted PDT. (A)** Chemical structures of PEG-b-PCL copolymer, CAR, and PhA. **(B)** Co-incorporation of PhA and CAR into PEG-PCL micelle. **(C)** Schematic presentation of photodynamic cancer therapy using PhA/CAR micelles: i. intravenous administration of drug-loaded micelles; ii. during blood circulation, PhA/CAR micelles is expected to be safe with minimal photodamage to non-target tissues induced by CAR-mediated singlet oxygen scavenging; iii. when the micelles were internalized by cancer cells, PhA and CAR molecules are released from the micelles and then become spatially isolated, resulting in PhA-induced singlet oxygen production and photokilling activity; iv. tumor site restored to health after PDT. Reproduced with permission from: [76].

Indeed, polymeric micelles are attractive carriers for improving PS delivery by enhancing aqueous solubility and retaining the PS payload in the blood for an extended period of time, thus allowing for sufficient EPR-mediated accumulation in the pathological area. However, PS-loaded micelles in blood circulation have been found to present a risk of skin photosensitivity, which could lead to damage of endothelial cells or neighboring blood-vessel cells. In order to reduce unwanted photoactivity at non-target sites, we have proposed a new concept for PS-loaded micelles with the ability to prevent singlet oxygen production during the post-treatment period (Figure 4) [76]. As a proof-of-principle of our strategy, PEG-PCL micelles co-loaded with PhA as the singlet oxygen generator and β-carotene (CAR) as the singlet oxygen scavenger were prepared. We observed that the CAR scavenger in the PhA/CAR micelles significantly diminished PhA-generated singlet oxygen through direct singlet oxygen scavenging in a physiological condition. However, tumor-cell-internalized PhA/CAR micelles exhibited remarkable phototoxicity because of the termination of the scavenging reactions by the intracellular disassembly of the micelle structure and the subsequent drug release. These findings suggest that PhA/CAR micelles could be a promising PDT agent with controllable PDT activity for cancer treatment. Our approach, which co-incorporates a singlet oxygen scavenger in combination with PS into micelles, is an attactive strategy that could permit more accurate PDT to minimize photodamage to non-target tissues, blood cells, or healthy tissues, thus making PDT a safer and more selective clinical technique.

While the polymeric micelle has been extensively investigated as a prospective platform for delivery of hydrophobic photosensitizing agent, it has also designed to delivery of a genetically encoded PS for photodynamic cancer treatment [77]. Genetically encoded PS is a recently reported optogenetic tool that enables local ROS production mediated by a fluorescent protein [78]. The unique feature of genetically encoded PS is that cancer cells bear a gene encoding phototoxic protein and produce protein in the adjusted cell compartment, consequently, being expressed in tumor cells rather than administered exogenously [79]. Therefore, the problems of associated with nonspecific PS accumulation in normal tissues could be addressed. Two fluorescent proteins having phototoxic properties are currently known, namely KillerRed (KR) and miniSOG (mini Singlet Oxygen Generator). KR is a dimeric green fluorescence protein (GFP)-like protein that can produce ROS by selective activation using a 585 nm of light [80]. Another phototoxic protein miniSOG is a small monomeric flavoprotein that can be excited at 448 nm and produce singlet oxygen with a high yield (0.47) [81]. However, for these fluorescence protein-based PSs, the ROS generation process only affects cells that express proteins, otherwise the cells will remain intact when they are prevented from uptake of the encoding therapeutic plasmids. In this respect, polymeric micelle could be utilized to deliver the protein-based PSs for improving the applicability of genetically encoded PS. In Muthiah’s report [77], a quantum dot (QDot)-encapsulated poly(2-hydroxyethyl aspartamide) grafted PEG and branched polyethylenimine (PPP) polymeric micelle has been prepared to deliver the KR plasmid to cancer cells. The quantum dot, CdSe/ZnS, was used as a light harvesting moiety that can facilitate the locally emission of green light and subsequently activation of the protein PS within cytoplasm. Intracellular activation of PPP-QDot/KR micelles using a blue light leads to efficient production of ROS; therefore, morphology changes, reduced metabolic activity, and apopotosis of PPP-QDot/KR-treated cancer cells are observed. In addition, by targeting the uptake of PPP-QDot/KR micelles to cancer cells, the cells can be selectively destroyed without affecting normal cells in terms of survival or proliferation. These findings suggest that delivery of the KR using polymeric micelles with multifunctional capacity can be an effective cancer treatment strategy.

### Activatable photosensitizer formulations based on polymer-PS conjugates for tumor site-specific PDT

Based on the mechanism of action of PDT, the direct photodamages on tumor cells highly depend on the in situ generation of cytotoxic singlet oxygen that causes the irreversible damages to pivotal biomacromolecules and other cellular components. Implementation of a controllable singlet oxygen generation with high selectivity and localization would lead to more reliable PDT with minimial phototoxicity to normal cells and hence enhanced efficacy for photodynamic cancer treatment. Above mentioned polymer-based nanocarriers have been considered as targeting platforms for PS delivery. These carrier systems have been demonstrated to show tumor-selective accumulation of PS due to the EPR effect that may improve the therapeutic efficacy of PDT. However, the EPR-based targeting strategies are usually incapable to restrict the localization of PS activation, which may be caused by the unexpected leakage of the PSs during systemic circulation [82]. Therefore, the control of tumor site-specific singlet oxygen production may be essential for avoiding associated side effects of PDT. An activatable PS formulation is a more sophisticated class for PS delivery, which can keep the photoactivity of PS dormant during systemic circulation but demonstrate phototoxicity only at the tumor site [83]. Generally, activatable PSs can be constructed by chemically linking PS with its activation controllers through stimuli-sensitive or cleavable linkers. The linkers between the PS and its activation controller can be specifically degraded by a variety of physicochemical and biological stimuli that exist solely or at very high levels in tumor sites, releasing PS in a photo-active form. PDT with activatable PSs is a promising therapeutic option since it can directly kill cancer cells with reduced side effects in non-target tissues [82–84]. However, current activatable PS formulations are often constructed with a low-molecular-weight system, such as PS-PS [85] or PS-quencher conjugate [86] system, which may suffer rapid elimination from the body and may possess multiple drug resistances, resulting in a short plasma half-life and low bioavailability. In order to address these shortcomings, the activatable formulations based on polymer-PS conjugates have been recently developed. Self-quenchable nanoparticles [48–52] formed by self-aggregation of PS-polymer amphiphilies and PS-polymer conjugate/quencher nanocomplexes [53–55] have been recently reported. These platforms not only possess the common characteristic features of low-molecular-weight activatable systems but also can be maintained stable in the circulatory system for an extended time period due to their appropriate size and surface properties, increasing the possibility for selective accumulation at tumor site through EPR effect.

### Self-quenchable nanoparticles

**Figure 5:**
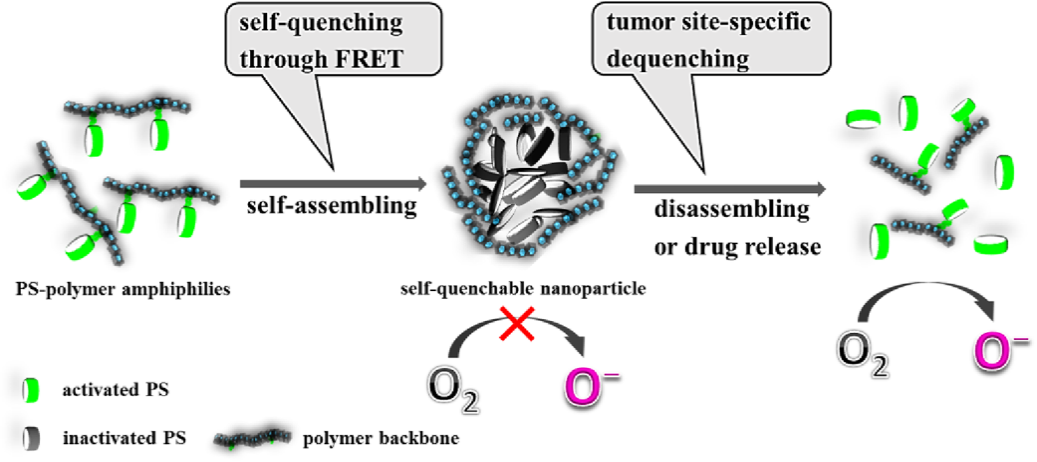
**A schematic diagram of self-quenchable nanoparticles.** The self-quenchable nanoparticles can incorporate and deliver the PS in a photodynamically inactive state and create active forms and produce cytotoxic effects only at the tumor site.

**Figure 6:**
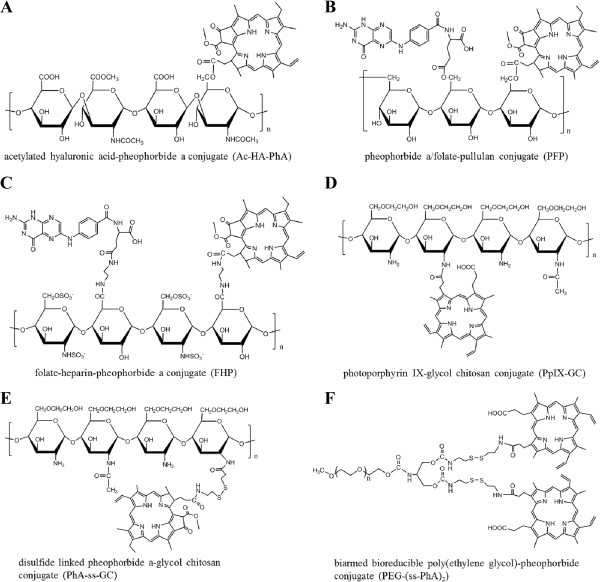
**Various of PS-polymer amphiphilies for preparation of self-quenchable nanoparticles: (A) acetylated hyaluronic acid-pheophorbide a conjugate (Ac-HA-PhA)** [48] **, (B) pheophorbide a/folate-pullulan conjugate (PFP)** [49] **, (C) folate-heparin-pheophorbide a conjugate (FHP)** [50] **, (D) photoporphyrin IX-glycol chitosan conjugate (PpIX-GC)** [82] **, (E) disulfide linked pheophorbide a-glycol chitosan conjugate (PhA-ss-GC)** [51] **, (F) biarmed bioreducible poly(ethylene glycol)-pheophorbide conjugate (PEG-(ss-PhA)**_**2**_ **)** [52] **.**

Some active PDT agents have been based on energy transfer between PSs by control of the aggregation/disaggregation (Figure 5). In this respect, PS-PS self-quenching through fluorescence resonance energy transfer (FRET) mechanism is the deactivation strategy involved in control of the photoactivity of the PSs. FRET is a nonradiative and distance-dependent energy transfer process that based on the interaction between the electronic excited states of two dye molecules in which excitation is transferred from a donor molecule to an acceptor molecule [83]. In recent, a variety of PS-polymer amphiphilies were synthesized for development of self-quenchable nanoparticles (Figure 6) [48–52]. When the hydrophobic PS molecules conjugated to the hydrophilic backbone polymers with an appropriate hydrophilic-hydrophobic balance, the amphiphilic nature of such polymer-PS conjugates could allow them to readily self-assemble to form nano-sized core-shell-structured nanoparticles in aqueous conditions, resulting in π-π-stacked self-aggregation among hydrophobic PS molecules in the NPs. Under light exposure, PS molecule promotes from its ground state to an excited singlet state, the neighboring PS molecule, as a FRET acceptor, may act on this excited singlet state before the PS enters its triplet state through energy transfer, resulting in the interruption of intersystem crossing. Thus, the pathway for singlet oxygen generation is blocked. As the result of efficient self-quenching, the desired characteristics of self-quenchable nanoparticles can allow the PS molecules to remain photodynamically inactive in the nanoparticulate formulation during systemic circulation to inhibit harmful singlet oxygen generation. In addition, the suppression can be rapidly reversed by molecular stimuli or environment conditions at the tumor site, which allows the PSs to restore the photoactivity and consequently produce cytotoxic species under light exposure.

In recent studies, natural polysaccharides have been popularly used for the development of self-quenchable nanoparticles due to their outstanding merits, such as high water solubility, good biocompatibility, a wide range of molecular weights, and an abundance of sources. Self-quenchable nanogels synthesized from acetylated hyaluronic acid-PhA (Ac-HA-PhA) conjugates were reported [48]. The Ac-HA-PhA amphiphilic conjugates readily formed 125 ~ 150 nm nanogels when degree of substitution of PhA molecules per glycosaminoglycan unit of AC-HA varied from 0.31 to 0.08. The resulting nanogels quenched both the PhA’s fluorescence and singlet oxygen generation through a hydrophobic interaction between PhA themselves. In tumor cells, the photoactivity of Ac-HA-PhA nanogel could be restored, due to the dequenching behavior induced by the enzymatic degradation of polysaccharide backbone, thereby producing singlet oxygen to kill tumor cells under light exposure. Another self-quenchable system based on pullulan/folate-PhA (PFP) conjugates was reported by the same group [49]. The enzyme-treated PFP self-quenchable nanogel showed fluorescence and singlet oxygen generation, which could not be observed in PBS due to the self-quenching effect between PS moieties. In the cell culture system, a dramatic increase in photoactivity induced by disintegration via lysosomal enzymes was observed. In addition, the PFP nanogel exhibited significant phototoxicity to tumor cells, while its cytotoxicity in darkness was negligible in the test range. After subcutaneously injection of PFP nanogels into the non-tumor-bearing Balb/C-nude mice, fluorescence did not immediately appear but was detected after 30 min and significantly increased for 12 h due to the dissociation and degradation-induced dequenching of the nanogels. Our group reported synthesis of heparin-PhA (HP) and folate-heparin-PhA (FHP) conjugates for preparation of self-quenchable nanoparticles for controllable PDT [50]. HP and FHP conjugates could self-assemble in aqueous media to form nano-sized particles with 130–170 nm in size. They displayed a self-quenching effect in PBS, while the generation of singlet oxygen dramatically increased in DMF where they exist as dissociated forms. In tumor cells, HP and FHP nanoparticles exhibited marked phototoxicity and were minimally dark-toxic without light treatment. Lee et al. has developed the protoporphyrin IX (PpIX) conjugated glycol chitosan (GC) nanoparticles as a self-quenchable system for PDT [87]. PpIX-GC nanoparticles showed the self-quenching effect with no fluorescence signal and phototoxicity under light exposure, which is due to the compactly crystallized PpIX molecules in the nanoparticles. The switchable photoacitivity of PpIX-GC nanoparticles attributes to dequenchability induced by disruption of the condensed nanoparticle structure at the harsh intracellular condition. After intravenous injection into HT-29 human colon adenocarcinoma tumor-bearing mice, they were observed to rapidly and significantly accumulate into the tumor tissue, and displayed strong fluorescence signals and significant therapeutic efficacy according to the effective in-body dequenching properties. By using GC as backbone polymer, we have reported cancer-cell specific PS nano-carrier by synthesizing PhA conjugated GC with reducible disulfide bonds (PhA-ss-GC) [51]. In order to enable quicker release kinetics and a more efficient activation mechanism of the PSs in the tumor cells, such a bioreducible nano-carrier system was especially interested, which might maximize the cytosolic dose of active PSs to achieve higher cytotoxicity, thereby enhancing the treatment efficacy. The relative fast intracellular degradation rate of PhA-ss-GC nanoparticles depends on that the thiol responsive disulfide linkers in the nanoparticles should be rapidly cleaved in the cytosol, as the concentrations of glutathione (GSH) in the cytosol (approximately 2–10 mM) are much higher than those in extracellular fluids (approximately 2–20 μM) [88]. The photoactivity of PhA-ss-GC nanoparticles in an aqueous environment was greatly suppressed by the self-quenching effect, which enabled the nanoparticles to remain photo-inactive and in a quenched state. However, after the cancer cell internalization, PhA-ss-GC nanoparticles rapidly restored their photoactivity, which is the result of the dissociation of the nanoparticular structure induced by reductive cleavage of the disulfide linkers. Compared to non-reducible PhA-GC nanoparticles with stable amide linkages, PhA-ss-GC nanoparticles presenting higher cytotoxicity with light treatment. In addition, the PhA-ss-GC demonstrated tumor specific targeting behavior through the EPR effect and enhanced tumor therapeutic efficacy compared to free PhA in tumor-bearing mice.

The above mentioned self-quenchable photosensitizing formulations basically have made up using natural polysaccharides as backbone polymers, but these matrix-forming materials have not been presently approved by Food and Drug Administration (FDA) as safe excipients for anticancer drug delivery, therefore, the development of clinically relevant formulations for controllable PDT is still a challenge. PEG is a nontoxic, nonimmunogenic, nonantigenic, and water-soluble polymer that is approved by the FDA as a safe excipient for ststemic delivery [65]. Utilizing PEG as a backbone polymer may promote the strategy regarding self-quenchable PS carrier system more close towards practical clinical application. In recent, we developed a bioreducible biarmed mPEG-(ss-PhA)_2_ conjugate for cancer-cell specific photodynamic therapy [52]. PhA molecules were chemically conjugated with biarmed linkages at one end of the mPEG molecule via disulfide bonds. As expected, the amphiphilic mPEG-(ss-PhA)_2_ conjugates readily self-assembled to form core/shell nanoparticles in the aqueous media. We observed that the photoactivity of mPEG-(ss-PhA)_2_ nanoparticles was significantly suppressed in the physiological conditions because of their self-quenching properties. However, when the NPs are internalized by cancer cells, the intracellular reduction-triggered cleavage of the disulfide bonds accelerates the dissociation of the nanoparticle structure and causes the PhA molecules to restore their photoactivity. The bioreducible activation mechanism of mPEG-(ss-PhA)_2_ NPs in cancer cells can efficiently maximize the cytosolic dose of active PSs to achieve high cytotoxicity under light exposure, thereby enhancing the treatment efficacy of photodynamic cancer treatment with reduced side effects.

### Polymer-PS conjugate/quencher nanocomplexes

**Figure 7:**
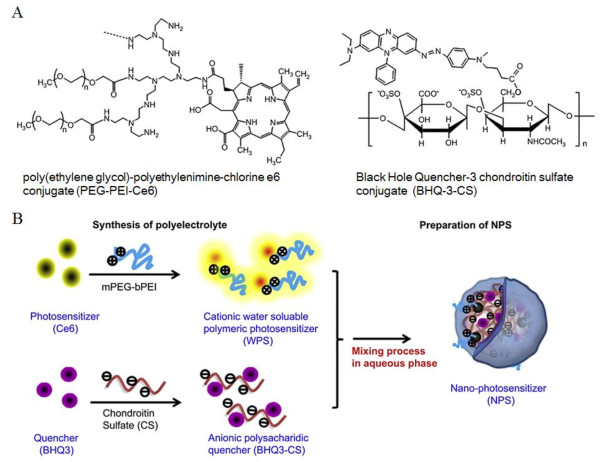
**Polyelectrolyte nanocomplex with controllable photoactivity prepared by ionic interactions between a cationic polymeric PS and an anionic polysaccharide quencher for PDT. (A)** Chemical structure of cationic water soluble polymeric PS (poly(ethylene glycol)-polyethylenimine-chlorine e6 conjugate (PEG-PEI-Ce6)) and anionic polysaccharide quencher (Black Hole Quencher-3 chondroitin sulfate conjugate (BHQ-3-CS)). **(B)** Synthesis of opposite charged polyelectrolyte and facile preparation of nano-PS in the aqueous phase. Reproduced with permission from: [53].

**Figure 8:**
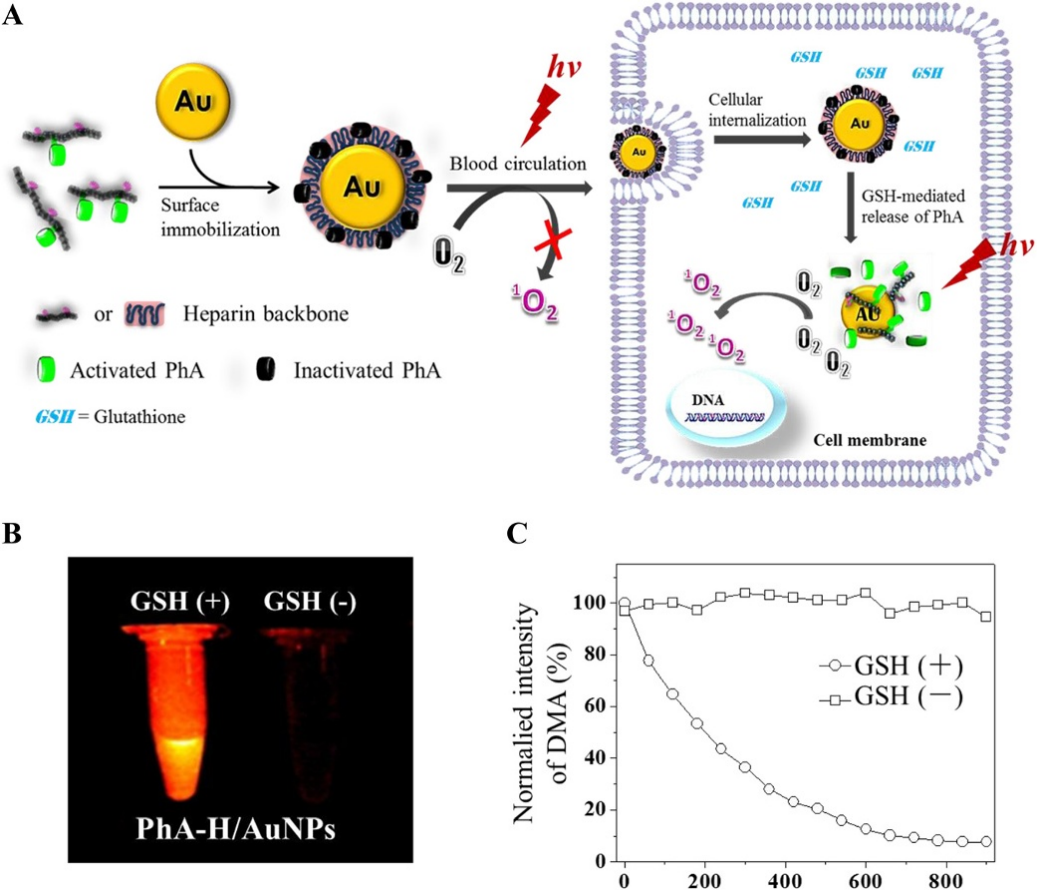
**Hybrid PhA-conjugated heparin/AuNP for cancer cell-specific PDT. (A)** Schematic diagram of formation of hybrid pheophorbide a/heparin-gold nanoparticle (PhA-H/AuNP) and GSH-mediated photoactivity: PhA becomes photoinactive when immobilized onto the AuNP surface. When PhA-H/AuNPs are internalized by cancer cells, GSH can trigger the release of PhA-H from AuNPs by cleavage of the gold-thiol linkages, causing the recovery of photoactivity of PS payloads. The activated PhA molecules can efficiently generate singlet oxygen to cause cancer cell destruction. **(B)** And **(C)**, GSH-mediated generation of fluorescence and singlet oxygen, respectively. Reproduced with permission from: [54].

Besides the self-quenching approach for control of the photoactivity of PSs, the specific deactivation of polymer-modified PSs can be achieved by PS-quencher complexation strategy. Generally, this strategy involves in synthesis of water-soluble PS-polymer conjugates, followed by binding to quencher molecules that act as FRET acceptors through covalent or physical bond. A polymeric PS with controllable photoactivity was prepared from a polyelectrolyte complex between cationic PEG-polyethylenimine-chlorine e6 conjugate (PEG-PEI-Ce6) and anionic Black Hole Quencher-3 chondroitin sulfate conjugate (BHQ-3-CS) (Figure 7) [53]. BHQ-3 was used as dark-quenching material that is able to deactivate the photoactivity of PS by FRET-based photoquenching when it was close neighbored with Ce6 in the nanoparticles. The nanoparticles clearly lost photoactivity by the intermolecular FRET effect in the aqueous phase. However, the quenched photoactivity was restored by the enzymatic degradation of BHQ-3-CS after esterase treatment. In the cell culture system, the rapid cellular internalization and the significant phototoxicity were observed. Recently, we have reported a new GSH-responsive hybrid nanoparticle, PhA-conjugated heparin/gold nanoparticle (PhA-H/AuNP), for controllable PDT (Figure 8) [54]. A thiolated water soluble polymeric PS, PhA-heparin, was synthesized and immobilized to the surface of AuNP via gold-thiol interaction. AuNP is a well-known quencher for efficient energy transfer based on FRET mechanism for excited dye molecules, which can quench the excited energy of fluorochromes even at a distance of around 40 nm. In our study, AuNP was used not only as a nanocarrier for loading the polymeric PS but also as a quencher material for deactivation of the surface attached PS. Furthermore, the release of PS payloads from the surface of AuNP triggered by GSH-mediated ligand exchange reaction can be expected to cause the effective dequenching processes and thus recovery of the photoactivity. As the result of quenching and dequenching behaviors of PhA-H/AuNP, we observed that the photoactivity was significantly suppressed in aqueous media, but instantaneously restored at the GSH-rich intracellular environment to generate a strong fluorescence signal together with active production of singlet oxygen species with light treatment. In vitro cell tests revealed marked phototoxicity and high intracellular uptake of PhA-H/AuNPs in contrast with free PhA. The PhA-H/AuNPs also exhibited a prolonged circulation characteristic, enhanced tumor specificity, and improved photodynamic therapeutic efficacy compared with free PhA in A549 tumor-bearing mice. As the follow up study, we further prepared a multifunctional hybrid nanoparticle system with a core/shell-structured magnetic Fe_3_O_4_/Au nanoparticle and a PhA-heparin surface layer as an activatable platform for PDT [55]. The novelty of the PhA-H/Fe_3_O_4_/Au hybrid nanoparticle lies in the fact of that unique and inherent surface property of gold shell: (i) suppression of photoactivity caused by the quenchability of the gold shell and (ii) restoration of photoactivity caused by GSH-mediated dequenchability of the surface-conjugated PS molecules. In addition, in vitro MRI studies reveal that the PhA-H/Fe_3_O_4_/Au nanoparticles could potentially serve as MRI contrast agents in cancer diagnosis and may be used to monitor the photodynamic treatment response used to accurately guide light irradiation.

In a summary, precise control of intracellular site of singlet oxygen production may be essential for efficient tumor cell destruction and reduce the side effect of PDT. Polymer-based activitable PSs are attractive formulations that may enhance therapeutic effect with reduced side effects. They have several advantages for efficient PS delivery: (1) the solubilization of hydrophobic PSs, (2) systemic delivery in a photoinactive form, (3) EPR-based tumor selectivity, (4) tumor site-specific generation of cytotoxic species under light exposure.

## Conclusion

PDT has emerged as one of the important therapeutic options in management of cancer. However, there are several shortcomings in the application of conventional PS-based PDT to the treatment of solid tumors. Most clinical applied PSs have limitations such as poor water solubility and low selectivity between tumor and normal tissues for clinical use. Physical encapsulation of PSs into polymer nanocarriers could overcome or reduce inherent limitations that the conventional PS formulations have. PS-encapsulated polymeric nanocarriers may act as a stable formulation for hydrophobic PS under physiological conditions and have an appropriate size for prolonged blood circulation time to deliver PSs to target sites. Moreover, self-quenchable nanoparticles formed by self-aggregation of PS-polymer amphiphilies and PS-polymer conjugate/quencher nanocomplexes have been proposed as a more sophisticated strategy to modulate the photoactivity PS. These PS-polymer conjugates-based formulations can not only engage in solubilization and selective accumulation but also can precisely control the photodynamical reactions only occurring at tumor site, thereby, maximizing the therapeutic efficacy and reducing the side effects related to classic PDT. Although the most of polymeric PS formulations are still undergoing testing in experimental animal models or pre-clinical trials, the above mentioned methodologies for PS delivery hold great potential to bring PDT to the forefront of oncological diseases treatment.
